# Anthocyanins Downregulate Lipopolysaccharide-Induced Inflammatory Responses in BV2 Microglial Cells by Suppressing the NF-κB and Akt/MAPKs Signaling Pathways

**DOI:** 10.3390/ijms14011502

**Published:** 2013-01-14

**Authors:** Jin-Woo Jeong, Won Sup Lee, Sung Chul Shin, Gi-Young Kim, Byung Tae Choi, Yung Hyun Choi

**Affiliations:** 1 Department of Biochemistry, Dongeui University College of Oriental Medicine, Busan 614-052, Korea; E-Mail: jinwooyo@nate.com; 2 Anti-Aging Research Center & Blue-Bio Industry RIC, Dongeui University, Busan 614-714, Korea; 3 Department of Internal Medicine, Institute of Health Sciences, Gyeongsang National University School of Medicine, Jinju 660-702, Korea; 4 Gyeongnam Regional Cancer Center, Gyeongsang National University Hospital, Jinju 660-702, Korea; 5 Department of Chemistry, Research Institute of Life Science, Gyeongsang National University, Jinju 660-701, Korea; E-Mail: sshin@gnu.ac.kr; 6 Faculty of Applied Marine Science, Cheju National University, Jeju 690-756, Korea; E-Mail: immunkim@cheju.ac.kr; 7 Division of Meridian and Structural Medicine, School of Korean Medicine, Pusan National University, Yangsan 626-870, Korea; E-Mail: choibt@pusan.ac.kr

**Keywords:** anthocyanins, BV2, anti-inflammatory activity, NF-κB, MAPK, Akt

## Abstract

Anthocyanins are naturally occurring polyphenols that impart bright color to fruits, vegetables and plants and have a variety of protective properties, which have generally been attributed to their antioxidant capacity. However, little is known about the molecular mechanisms underlying anti-inflammatory effects of anthocyanins related to neurodegenerative diseases. Therefore, we determined whether anthocyanins isolated from black soybean seed coats would inhibit pro-inflammatory mediators and cytokines in lipopolysaccharide (LPS)-stimulated murine BV2 microglial cells. Our results showed that anthocyanins significantly inhibited LPS-induced pro-inflammatory mediators, such as nitric oxide (NO) and prostaglandin E_2_, and pro-inflammatory cytokines including tumor necrosis factor (TNF)-α and interleukin (IL)-1β, without significant cytotoxicity. Anthocyanins also downregulated excessive expression of inducible NO synthase, cyclooxygenase-2, TNF-α, and IL-1β in LPS-stimulated BV2 cells. Moreover, anthocyanins inhibited nuclear translocation of nuclear factor-kappa B (NF-κB) by reducing inhibitor of NF-κB alpha degradation as well as phosphorylating extracellular signal-regulated kinase, c-Jun *N*-terminal kinase, p38 mitogen-activated protein kinase, and Akt. These findings suggest that anthocyanins may offer substantial therapeutic potential for treating inflammatory and neurodegenerative diseases accompanied by microglial activation.

## 1. Introduction

Microglia are resident macrophages in the central nervous system (CNS) and are thought to be a key mediator of brain disease and injury. Under normal conditions, these cells serve immune surveillance and host defense functions in the brain [1]. However, microglia become readily activated in response to injury, infection, or inflammation and are capable of producing a variety of pro-inflammatory mediators such as nitric oxide (NO), prostaglandin E_2_ (PGE_2_) and reactive oxygen species, pro-inflammatory cytokines, including interleukin-1β (IL-1β), IL-6 and tumor necrosis factor-α (TNF-α), and potentially neurotoxic compounds [2,3]. These factors are thought to be responsible for some of the deleterious effects of brain injuries and diseases, including ischemia, septic shock, Alzheimer’s disease, Parkinson’s disease, atherosclerosis, multiple sclerosis, and neural death [4,5]. Therefore, activated microglia could be a major cellular source of inflammatory and cytotoxic factors that cause neuronal damage in the CNS, and inhibiting these pro-inflammatory mediators and cytokines would be an effective therapeutic approach to mitigate the progression of neurodegenerative diseases.

Anthocyanins are flavonoids and water-soluble natural pigments responsible for the red, purple, and blue coloration in colored fruits and vegetables. They have been investigated for their potential benefits against cancer [6–9] as well as their antioxidative, cardioprotective [10,11] and anti-aging effects [12]. Moreover, anthocyanins have been reported for their neuroprotective and brain health benefits in humans and animals [13–16]. It is generally considered that these protective activities are related to the antioxidant properties of anthocyanins [7,9,17–19]. Kim *et al*. [20] isolated and fully characterized several anthocyanins from the seed coat of black soybean and investigated various pharmacological activities including anti-inflammatory and anti-diabetic properties [21–24]. However, few studies have been conducted on the effects of anthocyanins on microglia activation related to neurodegenerative disorders.

In this study, we investigated the effects of anthocyanins isolated from the black soybean seed coat on various lipopolysaccharide (LPS)-stimulated neurotoxic factors in murine BV2 microglia. We found that anthocyanins downregulated the production of pro-inflammatory mediators including NO and PGE_2_ and pro-inflammatory cytokines such as IL-1β and TNF-α suppressing LPS-induced activation of the nuclear factor-kappaB (NF-κB), phosphoinositide 3-kinase (PI3K)/Akt, and mitogen-activated protein kinases (MAPKs) signaling pathways.

## 2. Results and Discussion

### 2.1. Effects of Anthocyanins and LPS on BV2 Cell Viability

**Figure 1:**
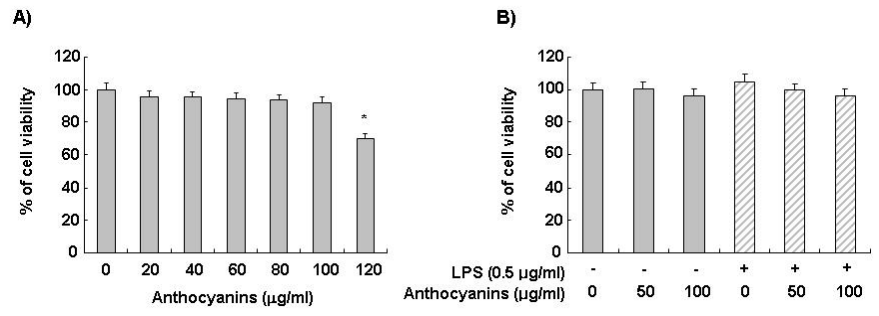
Effects of anthocyanins and lipopolysaccharide (LPS) on BV2 microglia viability. Cells were incubated with the indicated concentrations of anthocyanins for 24 h (**A**) or incubated with the indicated concentrations of anthocyanins or LPS (0.5 μg/mL) alone, or pre-treated with 50 and 100 μg/mL of anthocyanins for 1 h before incubation with LPS for 24 h (**B**). Cell viability was determined by the MTT assay. Data are expressed as mean ± SD of three independent experiments (*p < 0.05 *vs*. untreated control).

The MTT assay was performed at 24 h after treatment with the indicated concentrations of anthocyanins in the presence or absence of LPS to determine the effect of anthocyanins on BV2 cell viability. Anthocyanins alone at 20–100 μg/mL did not have a cytotoxic effect on BV2 cells. Anthocyanins in the presence of LPS (0.5 μg/mL) also did not show any cytotoxic effects on BV2 cell viability (Figure 1). Therefore, a concentration of anthocyanins within this range was applied in the remaining experiments.

### 2.2. Effects of Anthocyanins on LPS-Induced NO and PGE_2_ Production in BV2 Cells

**Figure 2:**
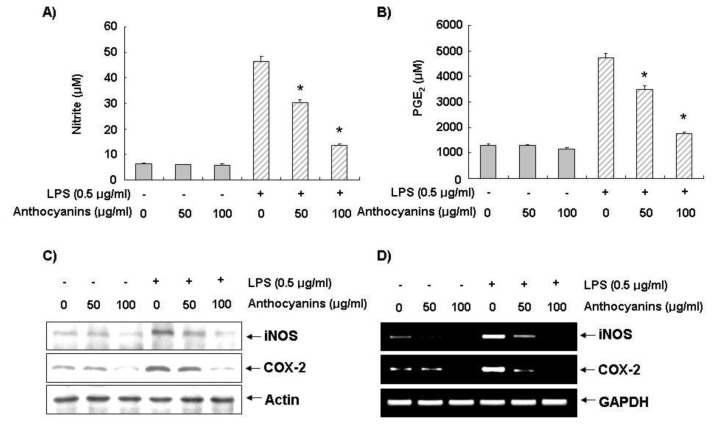
Inhibition of NO and PGE_2_ production, and iNOS and COX-2 expression by anthocyanins in LPS-stimulated BV2 microglia. Cells were pre-treated with 50 and 100 μg/mL of anthocyanins for 1 h before incubation with LPS for 24 h. (**A** and **B**) Culture supernatants were then isolated, and the amounts of NO (**A**) and PGE_2_ (**B**) production were determined. Data are expressed as mean ± SD of three independent experiments. *p < 0.05 indicates a significant difference from the value obtained for cells treated with LPS in the absence of anthocyanins. (**C**) Cells were lysed, and equal proteins were subjected to SDS-PAGE, followed by Western blotting using antibodies specific for murine iNOS and COX-2. (**D**) After LPS treatment for 6 h, total RNA was prepared for RT-PCR analysis of iNOS and COX-2 gene expression in LPS-stimulated BV2 cells. Actin and GAPDH were used as internal controls for the Western blot analysis and RT-PCR assays, respectively.

To evaluate the effect of anthocyanins on NO and PGE_2_ production, BV2 cells were stimulated with LPS (0.5 μg/mL) for 24 h after pre-treatment with 50 and 100 μg/mL of anthocyanins for 1 h. Cell supernatants were collected and assayed for NO and PGE_2_ production using the Griess reaction assay and ELISA. As shown in Figure 2A, treatment with LPS alone resulted in marked NO production from cells as compared with that generated by the control. However, pre-treatment with anthocyanins significantly repressed the levels of NO in LPS-stimulated BV2 cells in a concentration-dependent manner. In particular, 100 μg/mL of anthocyanins reversed LPS-induced NO production by >72%. Stimulating the cells with LPS also resulted in a significant increase in PGE_2_ production; however, treatment with anthocyanins decreased PGE_2_ production in a dose-dependent manner (Figure 2B).

### 2.3. Effects of Anthocyanins on LPS-Induced iNOS and COX-2 Expression in BV2 Cells

We performed Western blot analysis and RT-PCR to detect of protein and mRNA levels to examine whether inhibiting NO and PGE_2_ production by anthocyanins was associated with decreased levels of inducible NO synthase (iNOS) and cyclooxygenase-2 (COX-2) expression The Western blot data showed that treatment with LPS significantly increased iNOS and COX-2 protein expression at 24 h; however, anthocyanins markedly inhibited iNOS and COX-2 protein expression in LPS-stimulated BV2 microglia in a concentration-dependent manner (Figure 2C). Consistent with the results of the Western blot analysis, RT-PCR data indicated that treatment with LPS significantly increased iNOS and COX-2 mRNA expression after 6 h (Figure 2D). However, treatment with anthocyanins resulted in a significant decrease in iNOS and COX-2 mRNA expression. Taken together, these data indicate that anthocyanins inhibit upregulation of LPS-stimulated NO and PGE_2_ production by suppressing iNOS and COX-2 expression.

### 2.4. Effects of Anthocyanins on LPS-Induced TNF-α and IL-1β Production and Expression

**Figure 3:**
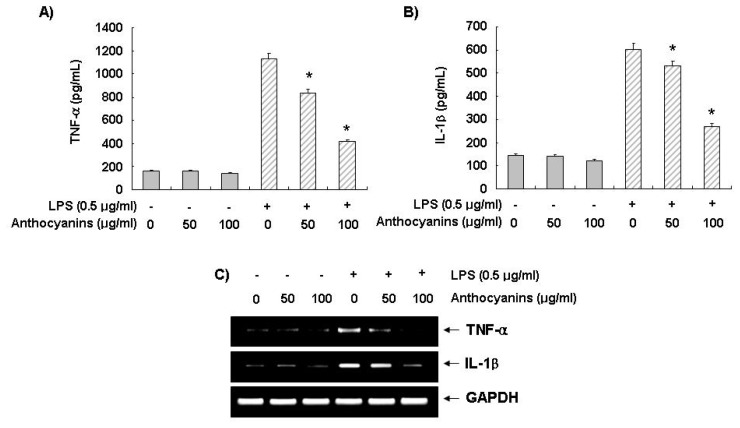
Effect of anthocyanins on LPS-stimulated TNF-α and IL-1β production and expression in BV2 microglia. BV2 cells were pre-treated with anthocyanins for 1 h before LPS treatment for 24 h. (**A** and **B**) Following 24 h incubation, the supernatants were taken, and amounts of TNF-α (**A**) and IL-1β (**B**) were measured by ELISA. Each value indicates the mean ± SD and is representative of results obtained from three independent experiments (*p < 0.05). (**C**) After 6 h incubation, levels of TNF-α and IL-1β mRNA were determined by RT-PCR. GAPDH was used as the internal control.

We next investigated whether anthocyanins regulated the production of proinflammatory cytokines, such as TNF-α and IL-1β, and their mRNA levels in LPS-stimulated BV2 cells. BV2 cells were pre-treated with 50 and 100 μg/mL of anthocyanins for 1 h, before LPS stimulation for 24 h, and the levels of TNF-α and IL-1β in the culture supernatant were determined by ELISA. As indicated in Figure 3A,B, untreated control or anthocyanins treatment alone sustained basal TNF-α levels. However, even though TNF-α and IL-1β production was upregulated significantly by LPS treatment, they were clearly suppressed after anthocyanins treatment in a concentration-dependent manner. In a parallel experiment, RT-PCR analyses were performed 6 h after LPS treatment to determine the effect of the anthocyanins on TNF-α and IL-1β gene expression. Consistent with TNF-α and IL-1β production, the RT-PCR data showed that anthocyanins suppressed TNF-α expression and IL-1β mRNA in LPS-stimulated BV2 cells (Figure 3C). These data indicated that anthocyanins regulate LPS-stimulated TNF-α and IL-1β release at the transcriptional level.

### 2.5. Effect of Anthocyanins on NF-κB Activity in LPS-Induced BV2 Microglia

**Figure 4:**
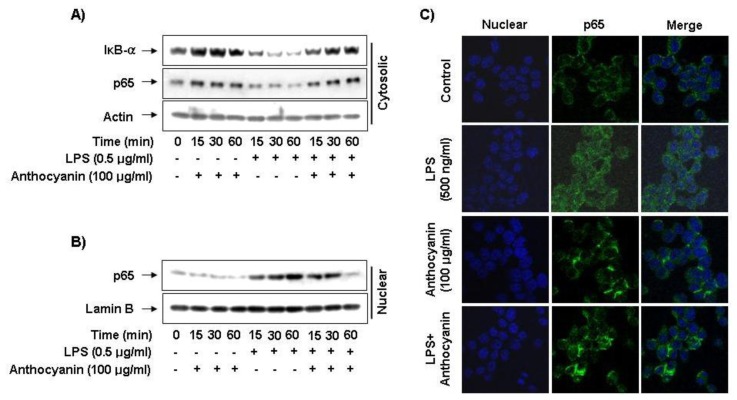
Effects of anthocyanins on NF-κB activity in LPS-stimulated BV2 microglia. (**A** and **B**) Cells were pre-treated with the indicated doses of anthocyanins for 1 h before LPS treatment for the indicated times. Cytosol (30 μg) or nuclear protein (30 μg) was subjected to 10% SDS-PAGE, followed by Western blotting using anti-NF-κB p65 and anti-IκB-α antibodies. Proteins were visualized using an enhanced chemiluminescence (ECL) detection system. Actin and lamin B were used as internal controls. (**C**) Localization of NF-κB p65 was visualized with fluorescence microscopy after immunofluorescence staining with NF-κB p65 antibody (green). Cells were stained with DAPI to visualize nuclei (blue). Results are representative of those obtained from three independent experiments.

Activation of NF-κB is closely related to regulation of iNOS, COX-2, TNF-α, and IL-1β genes in activated BV2 cells; therefore, we next investigated whether anthocyanins modulate the NF-κB activation in BV2 cells in response to LPS using Western blot and immunofluorescence microscopy analyses. The Western blot data showed a marked increase in the amount of NF-κB p65 in the nucleus after exposure to LPS alone; however, the LPS-induced p65 level in the nuclear fractions decreased in a concentration-dependent manner by anthocyanins pre-treatment (Figure 4A). In addition, inhibitor of NF-κB alpha (IκB-α) was markedly degraded at 15 min after LPS treatment; however, this LPS-induced IκB-α degradation was significantly reversed by anthocyanins (Figure 4B). Furthermore, the NF-κB shift to the nucleus in BV2 cells was analyzed using immunofluorescence staining and visualized with a fluorescence microscope to clearly understand the influence of anthocyanins on NF-κB p65 nuclear translocation. As indicated in Figure 4C, fluorescence images revealed that NF-κB p65 was normally sequestered in the cytoplasm (medium panel), and that nuclear accumulation of NF-κB p65 was strongly induced after stimulating BV2 cells with LPS (LPS panel). However, LPS-induced translocation of NF-κB p65 was completely abolished after pre-treating the cells with anthocyanins (LPS + anthocyanins panel). In addition, nuclear translocation of NF-κB p65 was not induced in cells after pre-treatment with anthocyanins alone in the absence of LPS stimulation (anthocyanins panel). These data indicated that anthocyanins treatment results in reduced NF-κB activity in LPS-stimulated BV2 microglial cells by suppressing p65 translocation.

### 2.6. Anthocyanins Inhibits LPS-Stimulated Phosphorylation of Akt and MAPKs in BV2 Microglia

**Figure 5:**
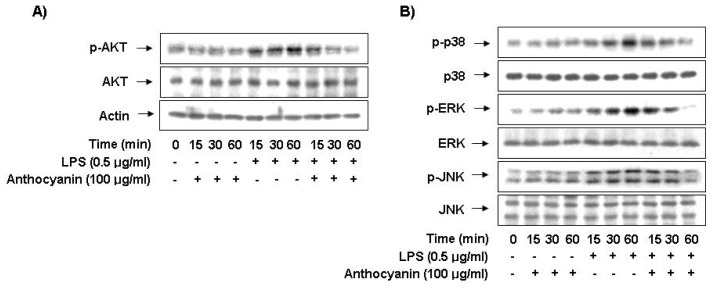
Effects of anthocyanins on Akt and MAPKs activation induced by LPS in microglia. BV2 cells were treated with the indicated does of anthocyanins for 1 h before LPS treatment for the indicated times. Total protein (50 μg) was subjected to 10% SDS-PAGE, followed by Western blotting using anti-Akt (**A**) and anti-ERK, anti-p38 MAPK, and anti-JNK antibodies (**B**). Results are representative of those obtained from three independent experiments. Actin was used as the internal control.

Recent findings have indicated that the Akt signaling molecule prompts NF-κB activation through the IκB degradation [25,26]. Therefore, we investigated the effect of anthocyanins on LPS-induced phosphorylation of Akt. As shown in Figure 5A, Akt phosphorylation increased markedly within 15 min after LPS stimulation; however, anthocyanins pre-treatment resulted in significant blockage of LPS-induced Akt phosphorylation, suggesting that Akt phosphorylation is involved in the inhibitory effect of anthocyanins on the LPS-induced inflammatory response in BV2 microglia.

Because MAPKs are the most important signaling molecules involved in regulating the synthesis and release of inflammatory mediators and cytokines by activated microglia [24,27], we next examined the effect of anthocyanins on LPS-induced activation of MAPKs, including extracellular signal-regulated kinase (ERK), c-Jun *N*-terminal kinase (JNK), and p38 MAPK. The Western blotting results indicated that LPS alone rapidly activated MAPKs within 1 h after stimulation. However, anthocyanins pre-treatment significantly inhibited phosphorylation of these kinases in LPS-stimulated BV2 microglia (Figure 5B), suggesting that anthocyanins are capable of disrupting the key signal transduction pathways activated by LPS in BV2 microglia.

### 2.7. Discussion

Microglia play a pivotal role in the innate CNS immune response, and serve as the first line of defense against invading pathogens by facilitating neuroprotection and repair processes [28,29]. However, abnormal activation of microglia induces a number of major cellular responses that play critical roles in the pathogenesis of inflammatory responses [1,30]. Microglia produce a variety of pro-inflammatory mediators, such as NO and PGE_2_, and pro-inflammatory cytokines, such as TNF-α and IL-1β, which play critical roles in severe neurodegenerative diseases [31,32]. In that regard, controlling microglial activation may have potential therapeutic options for treating various neurodegenerative conditions.

The pro-inflammatory mediators NO and PGE_2_ are the products of the inducible isoforms of iNOS and COX-2 enzymes, respectively [33]. NO is an important messenger molecule in a range of physiological and pathological processes, including vasodilation, neural communication, and host defense. However, overproduction of NO has also been associated with the initiation and maintenance of inflammation [34–36]. Similarly, PGE_2_ is also a well known inflammatory mediator derived from arachidonic acid via the action of COXs. Overproduction of PGE_2_ in response to growth factors, cytokines, and pro-inflammatory molecules is associated with up-regulation of COX-2. In particular, COX-2 is the predominant enzyme at sites of inflammation and edema [29]. There is accumulating evidence that confirms COX-2 as a potential therapeutic target for the treatment of inflammation and cancer [37,38]. In this study, anthocyanins from black soybean seed coats significantly inhibited LPS-induced release of the pro-inflammatory mediators NO and PGE_2_ from BV-2 cells. Interestingly, the inhibitory effects of anthocyanins on the LPS-induced release of pro-inflammatory mediators correlated with their abilities to suppress the expressions of their genes in BV-2 cells (Figure 2).

Chronic activation of microglia and consequent overproduction of pro-inflammatory cytokines are a histopathological hallmark of various neurological diseases. Among several cytokines, TNF-α and IL-1β are the main pro-inflammatory cytokines produced by activated microglia during inflammation in the CNS, and their excess production has been linked to many neurodegenerative disorders. They also play a pivotal role in the initiation and progression of severe neurodegenerative diseases as pleiotropic inflammatory cytokines [2,3,39]. These data indicate that regulating the expression of pro-inflammatory cytokines is a potential strategy to cure inflammatory diseases. Our data indicated that anthocyanins inhibited the LPS-induced TNF-α and IL-1β expression levels at the transcriptional level, which led to reduced production of TNF-α and IL-1β (Figure 3). These results suggest that anthocyanins may modulate the gene expression levels of TNF-α and IL-1β, which control their release.

Excess production of pro-inflammatory components in over-activated microglia may be a risk factor for initiating neurodegenerative onset via many cell signaling pathways. Among them, the nuclear transcriptional factor NF-κB is a key inflammation regulator due to its ability to induce transcription of pro-inflammatory genes, which are modulated by the binding of NF-κB to specific promoter regions [40,41]. NF-κB is normally located in the cytoplasm where it is complexed with the inhibitory IkB protein. In response to pro-inflammatory stimuli, IκB is phosphorylated and subsequently degraded, and NF-κB is released and translocated to the nucleus [42] where it promotes expression of inflammation-related genes. Involvement of the PI3K/Akt pathway in the expression of inflammatory mediators in microglia through NF-κB activation has been demonstrated [27,28,43]. Therefore, modulating NF-κB activity is considered a promising target for treating many neuropathologies, and we demonstrated marked blockage of LPS-stimulated degradation of IκB and nuclear translocation of NF-κB p65 by anthocyanins in BV2 microglia (Figure 4). Taken together, these results suggest that anthocyanins inhibit the expression of pro-inflammatory genes by suppressing LPS-induced NF-κB activity. Furthermore, anthocyanins significantly inhibited Akt activation in LPS-stimulated BV2 microglia, indicating that anthocyanins inhibit LPS-induced NF-κB activation by inactivating the PI3K/Akt signaling pathway.

## 3. Experimental Section

### 3.1. Reagents and Antibodies

LPS (*Escherichia coli* 026:B6), 3-(4,5-dimethylthiazol-2-yl)-2,5-diphenyl-tetrazolium bromide (MTT), and 4,6-diamidino-2-phenylindole (DAPI) were purchased from Sigma-Aldrich (St. Louis, MO, USA). Dulbecco’s modified Eagle’s medium (DMEM) containing l-glutamine (200 mg/L), fetal bovine serum (FBS) and other tissue culture reagents were purchased from Gibco-BRL (Grand Island, NY, USA). Reverse transcription polymerase chain reaction (RT-PCR) reagents were purchased from Promega (Madison, WI, USA). Nuclear and cytoplasmic extraction reagents (NE-PER^®^ Nuclear and Cytoplasmic Extraction Reagents) and the enhanced chemiluminescence (ECL)-detecting reagent were purchased from Pierce Biotechnology (Rockford, IL, USA). Antibodies against iNOS, COX-2, NF-κB p65, IκBα and phospho (p)-IκBα polyclonal antibodies were purchased from Santa Cruz Biotechnology (Santa Cruz, CA, USA). Antibodies against ERK, phospho (p)-ERK, p38 MAPK, p-p38 MAPK, JNK, and p-JNK were purchased from Cell Signaling Technology (Beverly, MA, USA). The antibody against β-actin was obtained from Sigma-Aldrich. Peroxidase-labeled goat anti-rabbit immunoglobulin and FITC-conjugated donkey anti-rabbit IgG were purchased from Amersham Co. (Arlington Heights, IL, USA) and Sigma-Aldrich, respectively. All other materials were purchased from Sigma-Aldrich (St. Louis, MO, USA).

### 3.2. Preparation of Anthocyanins

Anthocyanins isolated from seed coats of black soybean (*Glycine max* (L.) *Merr.*) were a generous gift from Dr. S.C. Shin (Department of Chemistry, Gyeongsang National University, Korea), and 100 mg/mL concentration stock solution was made by dissolving the anthocyanins in distilled water. For the isolation of anthocyanins, the seed coats of soybean accessions (200 g) were extracted for 24 h at 4 °C with methanol. The extraction was repeated three times. After concentration under reduced pressure, the extract was diluted to a total volume of 200 mL and partitioned against ethyl acetate (3 × 200 mL). The solution containing anthocyanins was concentrated to 100 mL. The solution was subjected to an Amberlite XAD-7 (Sigma-Aldrich) column and washed with deionized water and eluted with methanol containing 1% HCl. The solvent was vaporized under reduced pressure and the purple sticky solids dissolved in 50 mL of 30% aqueous methanol containing 1% HCl. The solution was applied to a column packed with Sephadex LH-20 (Amersham Biosciences, Sweden) and eluted using 30% aqueous methanol containing 1% HCl. Cyanidin-3-glucoside, delphinidin-3-glucoside, and petunidin-3-glucoside were isolated from Seed Coats of Black Soybean and used as anthocyanin source. The compositions of anthocyanin consisted of cyanidin-3-glucoside (72%), delphinidin-3-glucoside (20%) and petunidin-3-glucoside (6%).

### 3.3. Cell Culture and Cell Viability

The BV2 immortalized murine microglial cell line was provided by Dr. I.W. Choi (Inje University, Busan, Korea). The BV2 microglial cells were cultured in DMEM supplemented with 10% FBS, 100 U/mL penicillin, and 100 μg/mL streptomycin. Cells were maintained in a humidified incubator with 5% CO_2_. The cells were pre-treated with the indicated concentrations of anthocyanins for 1 h before the adding LPS (0.5 μg/mL). Cell viability was evaluated by the MTT reduction assay. In brief, cells (1 × 10^5^ cells/mL) were seeded and treated with various reagents for the indicated time periods. After treatment, the medium was removed, and the cells were incubated with 0.5 mg/mL MTT solution. After 3 h incubation at 37 °C and 5% CO_2_, the supernatant was removed and formation of formazan was measured at 540 nm with a microplate reader.

### 3.4. Nitrite Assay

NO levels in culture supernatants were measured by the Griess reaction. BV2 cells (5 × 10^5^ cells/mL) were plated onto 24-well plates and pre-treated with anthocyanins for 1 h before treatment with LPS (1.0 μg/mL) for 24 h. After LPS stimulation, 100 μL of conditioned culture medium from each sample was mixed with the same volume of Griess reagent (1% sulfanilamide in 5% phosphoric acid and 0.1% naphthylethylenediamine dihydrochloride) and then incubated at room temperature for 5 min. The absorbance was measured at 540 nm on a microplate reader. Nitrite concentration was calculated with reference to a sodium nitrite standard curve generated with known concentrations [44].

### 3.5. Measurement of PGE_2_ Production

BV2 cells were sub-cultured in 6-well plates (5 × 10^5^ cells/mL) and incubated with the indicated concentrations of anthocyanins in the presence or absence of LPS (0.5 μ/mL) for 24 h. One hundred microliters of culture-medium supernatant was collected for determination of PGE_2_ concentration by enzyme-linked immunosorbent assay (ELISA) Cayman Chemicals, Ann Arbor, MI, USA).

### 3.6. Measurement of IL-1β and TNF-α Production

The levels of IL-1β, and TNF-α, produced were measured with the ELISA kits (R & D Systems, Minneapolis, MN, USA) according to the manufacturer’s instructions. Briefly, BV2 cells (1 × 10^5^ cells/mL) were plated in 24-well plates and pre-treated with the indicated concentrations of anthocyanins for 1 h before treatment with 0.5 μg/mL LPS for 24 h. One hundred microliters of culture-medium supernatants was collected to determine IL-1β, and TNF-α concentrations by ELISA [45].

### 3.7. Isolation of Total RNA and Reverse Transcription Polymerase Chain Reaction (RT-PCR)

Total RNA was isolated using TRIzol reagent (Invitrogen, Carlsbad, CA, USA) according to the manufacturer’s instructions. One microgram of RNA was reverse-transcribed using M-MLV reverse transcriptase to produce cDNA. PCR was performed using a Mastercycler (Eppendorf, Hamburg, Germany) with the indicated primers. The resulting amplification products were separated electrophoretically on 1% agarose gels and visualized by ethidium bromide staining. Glyceraldehyde-3-phosphate dehydrogenase (GAPDH) was used as an internal control.

### 3.8. Western Blot Analysis

Cells were gently lysed for 30 min with lysis buffer (20 mM sucrose, 1 mM EDTA, 20 μM Tris–Cl, pH 7.2, 1 mM DTT, 10 mM KCl, 1.5 mM mgCl_2_, 5 μg/mL pepstatin A, 10 μg/mL leupeptin, and 2 μg/mL aprotinin) to prepare total protein Supernatants were collected and protein concentrations were determined using a Bio-Rad Protein Assay kit (Bio-Rad, Hercules, CA, USA). In a parallel experiment, cytoplasmic and nuclear proteins were extracted using nuclear and cytoplasmic extraction reagents according to the manufacturer’s protocol. For Western blot analysis, an equal amount of protein was subjected to electrophoresis on sodium dodecyl sulfate (SDS)-polyacrylamide gels and transferred to a nitrocellulose membrane (Schleicher & Schuell, Keene, NH, USA) by electroblotting. Blots were probed with the desired antibodies for 1 h, incubated with the diluted enzyme-linked secondary antibodies, and visualized by enhanced chemiluminescence according to the recommended procedure. Actin, ERK, and lamin B were used as internal controls for the total, cytosolic, and nuclear fractions, respectively.

### 3.9. Immunofluorescence Analysis

Cells were grown on glass coverslips for 24 h and then treated with 0.5 μg/mL LPS, which were either pre-treated or not pre-treated with anthocyanins for 1 h to detect of NF-κB p65 translocation. Cells were fixed in 3.7% paraformaldehyde, treated with 0.2% Triton X-100, and blocked with 2% BSA. The cells were then sequentially incubated with anti-NF-κB p65 antibody, FITC-conjugated donkey anti-rabbit IgG, and DAPI solution (2.5 μg/mL), and examined using a fluorescence microscope (Carl Zeiss, Oberkochen, Germany).

### 3.10. Statistical Analyses

Data represent means ± standard deviations. Statistical significance was determined using an analysis of variance, followed by Student’s *t*-test. A *p* < 0.05 was accepted as statistically significant.

## 4. Conclusions

In summary, our data demonstrated that anthocyanins significantly suppressed the release of pro-inflammatory mediators and cytokines by blocking the NF-κB pathway, and activating PI3K/Akt and MAPKs in LPS-stimulated BV2 microglia, without cytotoxicity. Although further study using animal models is necessary to determine whether anthocyanins show an anti-inflammatory effect *in vivo*, our data suggest that anthocyanins may provide a beneficial effect for treating inflammatory and neurodegenerative damage induced by microglial activation.
